# Elevation of soluble major histocompatibility complex class I related chain A protein in malignant and infectious diseases in Chinese patients

**DOI:** 10.1186/1471-2172-13-62

**Published:** 2012-11-26

**Authors:** Xiaoxin Jiang, Ju-Fang Huang, Zhi Huo, Qiuqui Zhang, Yan Jiang, Xiaoping Wu, Yanwen Li, Guanmin Jiang, Leping Zeng, Xiao-Xin Yan, Ping Yu, Renxian Cao

**Affiliations:** 1 The First Affiliated Hospital, Nanhua University, Hengyang, 421001, China; 2 Department of Immunology, Xiangya School of Medicine, Central South University, Changsha, Hunan, 410078, China; 3 Department of Anatomy and Neurobiology, Xiangya School of Medicine, Central South University, Changsha, Hunan, 410013, China

**Keywords:** MHC, sMICA/B, NKG2D, Cancer diagnosis, Serum

## Abstract

**Background:**

Elevation of soluble major histocompatibility complex class I chain-related gene A (sMICA) products in serum has been linked to tissue/organ transplantation, autoimmune diseases and some malignant disorders. Cells infected by microbiological pathogens may release sMICA, whereas less is known whether and to what extent serum sMICA levels may change in infectious diseases.

**Methods:**

The present study determined serum sMICA levels by enzyme-linked immunosorbent assay (ELISA) in a southern China population, including patients (n = 1041) suffering from several types of malignant and infectious diseases and healthy controls (n = 141).

**Results:**

Relative to controls, serum sMICA elevation was significant in patients of hepatic cancer, and was approaching statistical significance in patients with lung, gastric and nasopharyngeal cancers. sMICA elevation was also associated with some bacterial (Enterobacteriaceae, Mycobacterium tuberculosis, non-fermenting Gram-negative bacteria and Gram-positive cocci), viral (hepatitis B and C) and the Microspironema pallidum infections.

**Conclusion:**

Serum sMICA levels may be informative for the diagnosis of some malignant and infectious diseases. The results also indicate that microbiological infections should be considered as a potential confounding clinical condition causing serum sMICA elevation while using this test to evaluate the status of other disorders, such as cancers, host-graft response and autoimmune diseases.

## Background

A host organism can mount immune responses to “foreign” antigens during tissue transplant, against infectious pathogens and under autoimmune conditions. The major histocompatibility complex (MHC) or human leukocyte antigen (HLA) genes located in chromosome 6 encode the classical class I gene products that are involved in such immune responses. Thus, MHC class I (HLA-A, -B and -C) and class II (HLA-DR, -DQ and -DP) genes produce antigen-presenting molecules that stimulate CD8+ and CD4+ T cells activation. MHC class III or central MHC proteins perform various immune functions by participating complement and cytokine activities [1-4].

The human major histocompatibility complex class I chain-related genes (MIC) are lately discovered genes located on chromosome 6 in the region encoding the classic MHC products, This set of genes encodes protein products performing distinct immune functions than antigen presentation. The MIC region consists of 7 loci encoding two functional genes, namely the human major histocompatibility complex class I chain-related gene A (*MICA*) and B (*MICB*), with the remainder (*MICC-MICG*) being pseudogenes [5,6]. Specifically, the MICA gene is about 11.7 Kb in length, and is transcribed into a 1382 bp mRNA that has 6 exons separated by 5 introns. Mature MICA polypeptide consists of 383 amino acid residues (43 Kda), containing a leading sequence translated from exon 1, the α1 to α3 structural domains encoded by exon 2 to 4, and a transmembrane domain and a cytoplasmic domain that are determined by exons 5 and 6, respectively. The transmembrane domain encoded by exon 5 is rich of GCT microsatellite repeats, resulting in a great polymorphism. The MICB gene is organized in a similar manner to that of MICA, and shares ~91% base-pair sequence with the latter. Evidence suggests that the MICB gene also exhibits a certain degree of polymorphism [6-12]. MICA and MICB, including their polymorphism conditions, may relate to the susceptibility to cancer and infectious diseases among individuals, although the underlying mechanisms remain less clear at the present [13-23].

In addition to their membrane-bound forms, MICA and MICB can be released from the surface of tumor and infected cells following proteolytic cleavages, yielding soluble MICA (sMICA) and MICB (sMICA) in serum. By binding to NKG2D receptor, soluble MICA/B molecules may block the activation of effector lymph cells by MICA/B, thereby facilitate the escape of tumor or infected cells from immunosurveillance [1,2,24-30]. In patients with some types of cancers, serum levels of sMICA/B are elevated, whereas the NKG2D expression on NK and/or CD8^+^ T cells are downregulated. As such, soluble MICA/B in the circulation may therefore play an important biological role in modulating immune response [1,25,26].

sMICA/B have been used as potential biomarkers for assessing chronic graft-host immune response as well as the status of some malignant diseases [1,2,17,18,26]. Specifically, serum sMICA is elevated in patients with various carcinomas including gastrointestinal, lung, hematological, gynecological and urological cancers, with the extent of change reported to correlate with disease severity and/or metastasis status [24-43]. Other studies demonstrate serum sMICB elevation being of diagnostic value in cancer patients [34-43]. It should be noted that moderate increase of serum MICA/B has been demonstrated in some benign conditions, including non-malignant tumors and chronic inflammation [36,44-50]. However, association studies on sMICA/B alteration in various infectious disorders are scarce.

In the present study, we measured serum sMICA levels by enzyme-linked immunosorbent assay (ELISA) in a large patient population from Hunan Province of China. Subjects included patients with several types of clinically diagnosed cancers, bacterial and viral infections, and healthy controls. The extent of sMICA elevation and diagnostic value, i.e., sensitivity and specificity by receiver operating characteristic (ROC) analysis, were evaluated for each disease entity. Our results point to a modulation of sMICA in malignant as well as nonmalignant disorders, which might be of clinical implications for disease diagnosis and differential diagnosis.

## Methods

### Patients and healthy donors

The present human subject study was approved by the ethics committee of Nan-Hua (Southern China) University; with pre-informed consent obtained from all participants. Patients (n = 1041) were enrolled into the first affiliated hospital of the University from October, 2007 to December, 2009. Healthy controls (n = 141) were out-patients who received routine physical examination during the same period. All participants were Han Chinese. The general demographic information is summarized in Table 1. The malignant tumor group included 495 cases suffered from liver (n = 141), lung (n = 70), gastric (n = 77), intestine (n = 58), breast (n = 57), cervical and ovary (n = 46) and nasopharyngeal (n = 21) carcinoma, as well as lymphoma (n = 25). The bacterial infection group (n = 146) included patients with symptomatic infections by enterobacteriaceae (n = 40), non-fermenting Gram-negative bacteria (n = 39), Mycobacterium tuberculosis (n = 55) and Gram-positive cocci (n = 12). The viral infection group included 344 cases with clinical symptoms caused by hepatitis virus B (n = 74), hepatitis virus C (n = 94), and other viruses (n = 176) including herpes simplex virus, Coxsackie virus, Epstein-Barr virus and cytomegalovirus Fourteen and 42 cases were enrolled into the Candida albicans and Microspironema pallidum infection groups, respectively. Diagnoses were established based on clinical history, imaging examination and pathological evaluation for tumor patients [including by the TNM (tumor, node, and metastasis) staging system]. For all infectious disease patients, the diagnoses were also confirmed by serological tests and/or microbiological cultures using blood and/or body fluids.

**Table 1:** Demographic characteristics of the patients and healthy controls

**Subject groups**	**Number of cases**	**Sex (male/female)**	**Age (years) mean ± S.D.(range)**
Carcinoma	495	306/189	53.4 ± 13.0 (12-89)
Bacterial infection	146	103/43	52.6 ± 22.0 (0.1-91)
Candida albicans infection	14	3/11	66.2 ± 22.0 (0.2-90)
Virus infection	344	217/127	41.6 ± 21.9 (0.2-88)
Microspironema pallidum	42	24/18	37.2 ± 16.1 (1-69)
Healthy controls	141	81/60	44.0 ± 11.4 (22-70)

### Measurement of serum sMICA by ELISA

The capture antibody was a monoclonal antibody to human sMICA (mAbAMO-1, IgG1, BAMOMAB GmbH, Habsburgerstrasse, Germany). A total of 100 μl buffer containing the antibody at a concentration of 5 ng/ml was loaded into each well of 96-well plates. After antibody coating overnight at 4°C, the plates were washed 3 times with 0.01 M phosphate-buffered saline (PBS) containing 3% Tween-20 (PBST). To block non-specific binding, the plates were incubated with PBS containing 15% bovine serum albumin (BSA) at 37°C for 2 hours, and then rinsed with PBST once. Loading samples were mix of human serum and PBS containing 15% BSA, at a 1 to 2 ratio. For each case, 100 μl sample mix was loaded in each well of the plates, incubated at 37°C for 2 hours. The plates were then incubated with the reporter antibody anti-MICA 6B3 (monoclonal IgG2α, see [17,18]) at 1 μg/ml (100 μl) at 37°C for 2 hours, and with horseradish peroxidase (HRP) conjugated goat anti-rat IgG antibody (100 μl/well, 1:2000) at 37°C for 2 hours. Binding signal was visualized using tetramethyl benzidine (TMB) as a chromogen, with optic absorbance values measured at 450 nm (RT-6000, Rayto Life and Analytical Sciences Co. Ltd., USA). Recombinant human sMICA (MICA*008, see [17,18]) was prepared at titered concentrations (0.156 ng/ml, 0.31 ng/ml, 0.62 ng/ml, 1.25 ng/ml, 2.5 ng/ml, 5.0 ng/ml), and assayed in parallel with serum samples. All samples were assayed in triplicate.

### Data and statistical analysis

Concentrations of sMICA were calculated according to the standard curve derived from MICA*008 references. Data were expressed as mean ± SD, and subjected to One-way ANOVA test using the Prism 4.02 software (GraphPad Software, Inc., San Diego, CA, USA). The significant level was set at P < 0.05. To assess the diagnostic value of the assay, the sensitivity/specificity receiver operating characteristic (ROC) curve was generated with Excel. Cut-off level was set at the 95th percentile to a given testing group. Area under the ROC curve (AUC) (ranging from 0.5-1) was established to estimate the diagnostic value. According to clinical statistics, AUC values from 0.5 to 0.7, 0.7 to 0.9, and >0.9 are suggestive of low, intermediate and high diagnostic significance, respectively.

## Results

### Serum levels and diagnostic impact of sMICA in malignant diseases

**Figure 1:**
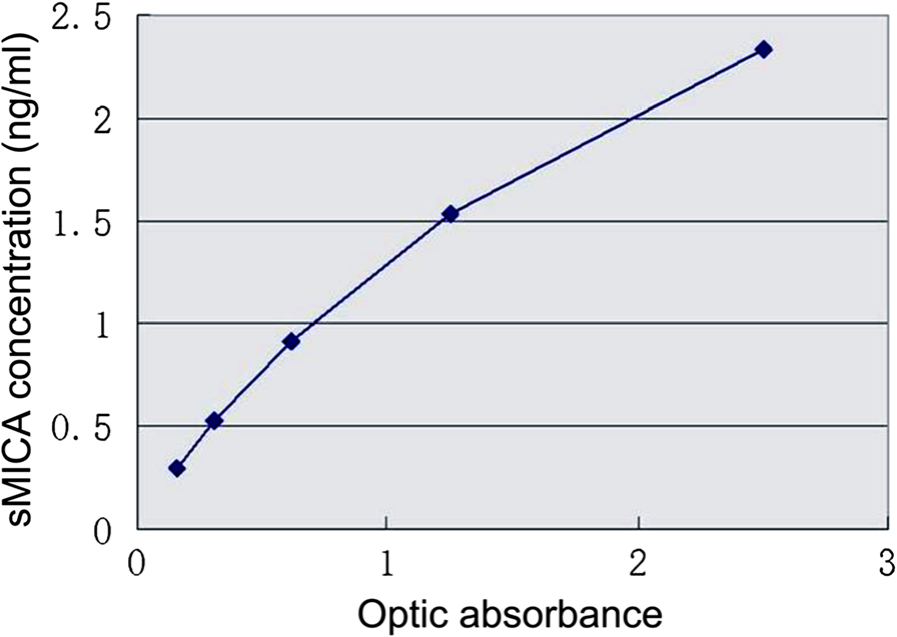
**Example of the standard curve for serum soluble Major histocompatibility Complex class I related chain A (sMICA) generated by Sandwich ELISA in a typical assay.** Concentrations of the recombinant MICA*008 are plotted against the readings of optic absorbance. A liner relationship exists between the values of optic absorbance and concentrations of the MICA*008. The values are averaged from duplicated loadings at each concentration.

**Figure 2:**
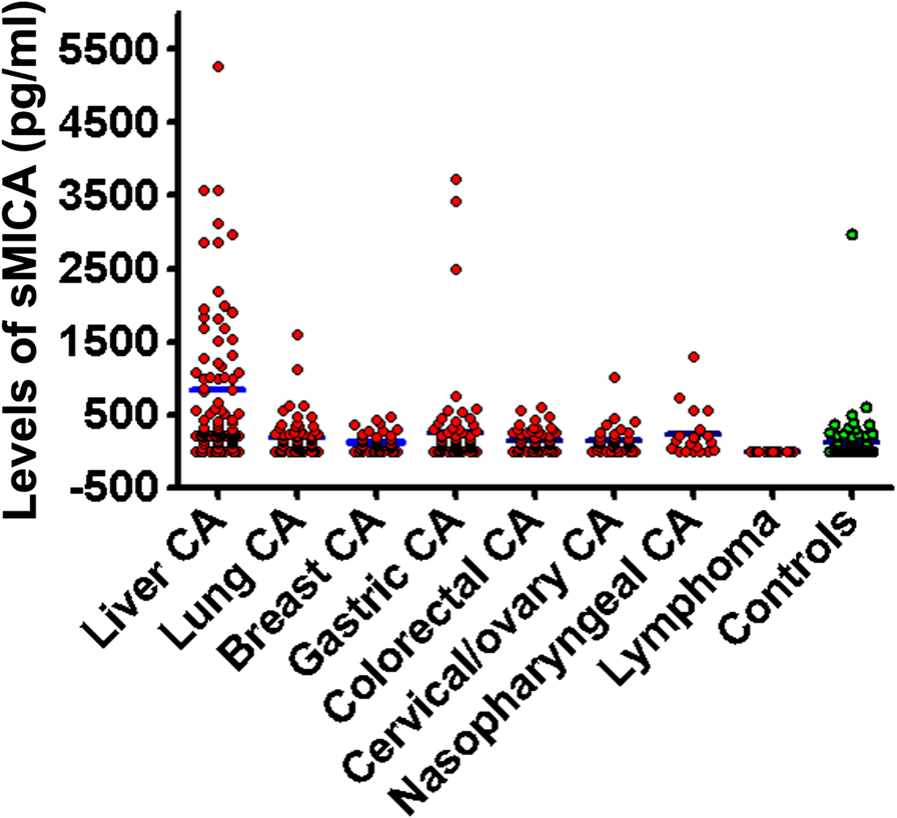
**Dot graph showing the distribution and means (blue bars) of serum sMICA concentration (pg/ml) among cancer patients (red) relative to healthy control (green) groups.** The liver cancer group shows the highest mean, with the levels of sMICA remarkably elevated among some individuals. Each dot represents an individual case, referring to Table 2 for case numbers in each group and statistics.

A linear relationship between sMICA concentrations and optic absorbance readings was established by the calibration assays using the recombinant human sMICA (Figure 1). Accordingly, serum levels of sMICA were obtained from the patient and control groups. Overall, patients with liver cancer had the highest levels of sMICA (743.4 ± 110.8 pg/ml) relative to other types of cancers and healthy controls (168.5 ± 56.7, pg/ml) (Table 1). Patients with gastric cancer ranked the second highest elevation of sMICA levels among the cancer groups (264.4 ± 524.8 pg/ml). In contrast, serum levels of sMICA in patients with female reproductive system tumors and malignant lymphoma (140.9 ± 137.6 pg/ml and 162.5 ± 116.1 pg/ml) appeared to be comparable to that in control (Figure 2, Table 2). One-way ANOVA analysis showed a significant difference among the cancer and control groups (P < 0.0001, F = 80.53, df = 8, 627). Posthoc tests indicated that the levels of sMICA in the liver cancer group were significantly different relative to the control (P < 0.0001) as well as the remaining cancer groups (P < 0.0001). The differences between the gastric cancer (P = 0.075) relative to control groups and between nasopharyngeal cancer (P = 0.087) relative to control groups, were approaching statistical significance (Table 2).

**Table 2:** Levels of soluble MICA in different cancer patients

	**Number of cases**	**Sex (male/female)**	**Age (years) mean ± SD (range)**	**sMICA (pg/ml)**	**P (vs controls)**
Liver cancer	141	118/23	52.7 ± 11.2(24-75)	743.4 ± 110.8	<0.0001
Lung cancer	70	54/16	59.5 ± 10.5(39-79)	192.3 ± 258.2	0.095
Breast cancer	57	0/57	50.0 ± 11.5(29-72)	162.5 ± 116.1	>0.05
Gastric cancer	77	63/14	52.4 ± 12.4(20-78)	264.4 ± 524.8	0.075
Colorectal cancer	58	39/19	58.8 ± 16.3(13-89)	176.4 ± 147.9	>0.05
Cervical/ovary cancer	46	0/46	46.3 ± 8.9(23-70)	168.3 ± 173.3	>0.05
Nasopharyngeal cancer	21	19/2	47.1 ± 10.9(30-74)	238.2 ± 318.6	0.087
Lymphoma	25	13/12	48.2 ± 13.6(17-70)	140.9 ± 137.6	>0.05
Controls	141	81/60	44.0 ± 11.4(22-70)	168.5 ± 56.7	

**Figure 3:**
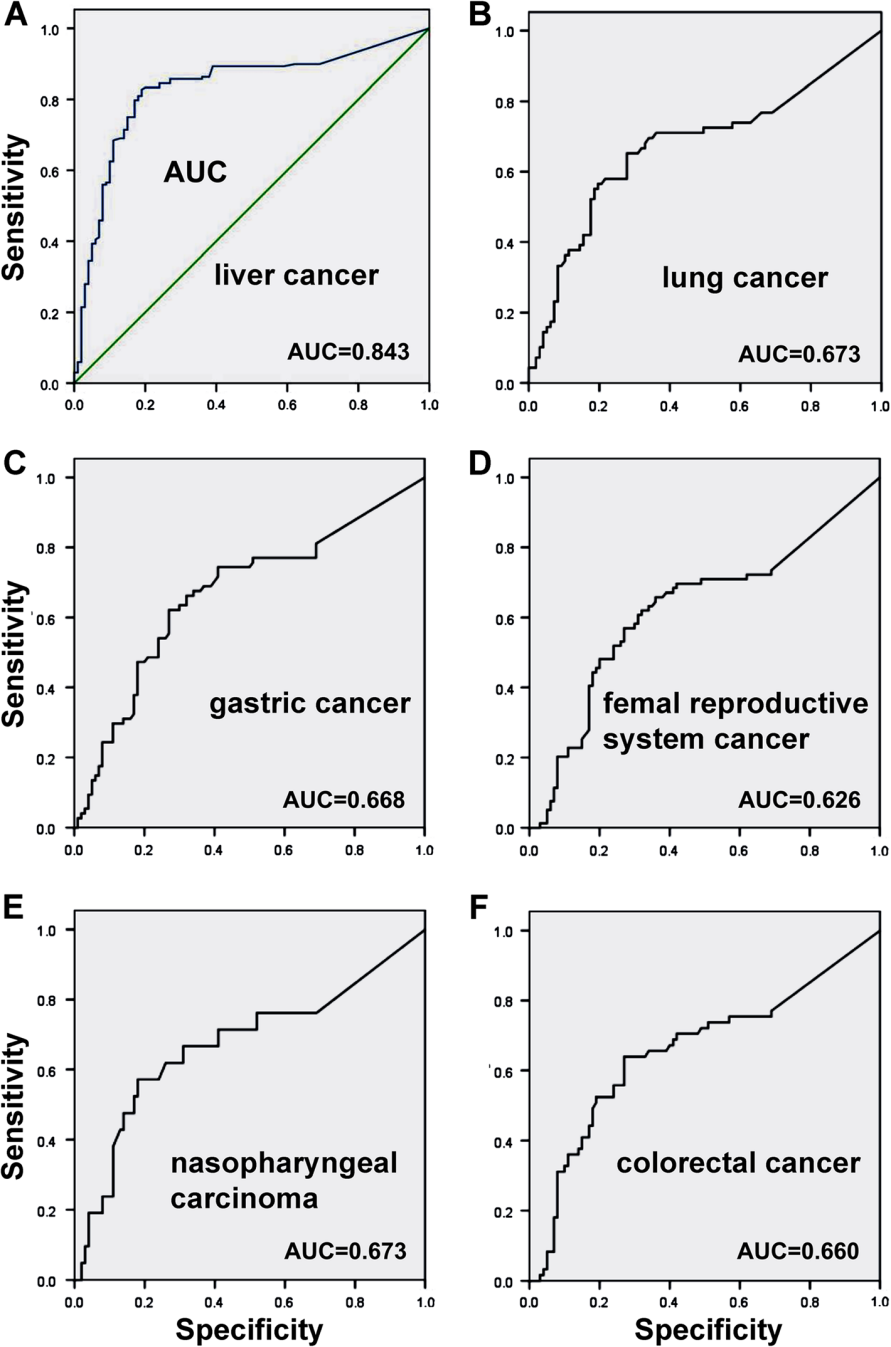
**Receiver operating characteristic (ROC) curves illustrating the diagnostic impact of serum sMICA levels in different malignant tumors, as determined by the values of area under the ROC curve (AUC).** The data indicate that test has the highest diagnostic value (AUC = 0.834) among patients of hepatic cancer relative to other malignant tumors.

The diagnostic impact of sMICA in the above cancer groups was analyzed by ROC curve fitting (Figure 3). The AUC value was 0.843 for hepatic cancer patients, which was of high diagnostic value (Figure 2A). The AUC values were 0.673, 0.668, 0.626, 0.673 and 0.660 for lung, gastric, female reproductive, nasopharyngeal and colorectal cancers, respectively. Thus, the AUC values of the non-hepatic malignant groups appeared to be of a relatively low diagnostic impact (Figure 3B-F).

Given the potential high diagnostic implication of sMICA among the liver cancer patients, we further analyzed whether serum sMICA levels were correlated to TNM staging and the pathological grade of the tumor. Levels of sMICA were 389.6 ± 153.3, 712.3 ± 108.6, 883.7 ± 133.2 and 699.1 ± 212.5 pg/ml in the subgroups with hepatic cancers classified as T1, T2, T3 and T4 stages, respectively (Table 3). Among all liver cancer patients, about 4.3%, 23.4%, 59.5% and 12.8% of the cases had the cancers graded as T1, T2, T3 and T4 clinical stages, respectively. The levels of sMICA were significantly higher for T1-T4 groups relative to controls (P < 0.0001, F = 655.8, df = 4,277). Posthoc tests indicated statistical differences for the T1 relative to T2, T3 and T4 subgroups (P < 0.001 for all subgroups), the T2 relative to T3 subgroups (P < 0.001) and the T3 relative to T4 subgroups (P < 0.001). However, while the data were analyzed against the pathologically defined differentiation grading, no statistical differences were found between the high, intermediate and low differentiation subgroups (Table 3).

**Table 3:** Serum sMICA levels relative to hepatic cancer staging

	**Tumor grading**	**Case subgroups**	**sMICA (pg/ml)**	**P (relative to T1)**
TNM staging	T1	4.3% (6)	389.6 ± 153.3	
	T2	23.4% (33)	712.3 ± 108.6	<0.0001
	T3	59.5% (84)	883.7 ± 133.2	<0.0001
	T4	12.8% (18)	699.1 ± 212.5	<0.0001
Pathological Differentiation	high	56.1% (79)	718.9 ± 113.6	>0.05
	Intermediate	33.3% (47)	753.5 ± 132.1	>0.05
	low	10.6% (15)	840.8 ± 232.2	>0.05

### Serum levels and diagnostic impact of sMICA in infectious diseases

**Figure 4:**
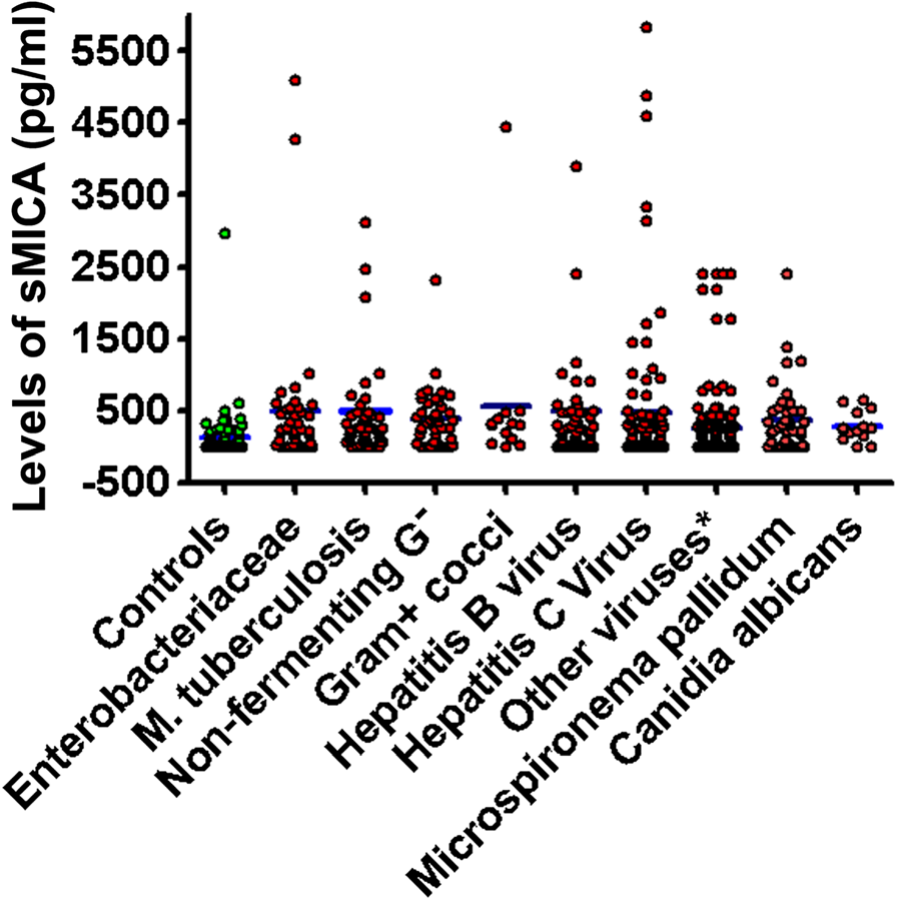
**Dot graph plots the distribution and means (blue bars) of serum sMICA concentration (pg/ml) among patients suffering various microbial infections (red), relative to healthy controls (green).** Each dot represents an individual case. Please refer to Table 4 for sample sizes and statistical analysis information.

We analyzed serum sMICA levels among patients suffered from several bacterial (enterobacteriaceae, non-fermenting Gram-negative bacteria, Gram-positive cocci, M. tuberculosis, Microspironema pallidum), viral (hepatitis B, hepatitis C and other viruses) and fungi (Canidia albicans) infections relative to the healthy controls, as detailed in Table 4. All infections were clinically established, with the diagnoses also confirmed by microbiological and serological laboratory tests. A significant difference existed among the infectious disease and control groups by one-way ANOVA (P = 0.0001, F = 21.88, df = 9, 677). Specifically, levels of sMICA were elevated among all infectious diseases, except for the Canidia albicans and “other viruses” groups, relative to controls as indicated by posthoc paired comparison (Figure 4; Table 4). Of note, the hepatitis B and C virus infection groups showed significant sMICA elevation relative to the “other viruses” group (P < 0.001). In sum, a certain extent of elevation of serum sMICA levels appeared to be associated with most bacterial infections as well as hepatitis B and C. In contrast, levels of sMICA in patients with Canidia albicans fungi infection appeared to be comparable to that in normal controls.

**Table 4:** Levels of soluble MICA in infectious diseases patients

**Pathogens**	**Number of cases**	**Sex (male/female)**	**Age (year) mean ± S.D. (range)**	**sMICA (pg/ml)**	**P (vs controls)**
Enterobacteriaceae	40	26/14	59.2 ± 22.1(1-91)	492.2 ± 295.9	<0.001
M. tuberculosis	55	41/14	56.1 ± 19.0(2-88)	493.9 ± 256.4	<0.001
Non-fermenting Gram-negative bacteria	39	26/13	49.4 ± 22.1(5-90)	384.1 ± 416.2	<0.001
Gram-positive cocci	12	10/2	38.9 ± 21.9(2-84)	542.3 ± 382.9	<0.001
Hepatitis B virus	74	45/29	44.1 ± 16.6(15-87)	472.4 ± 103.6	<0.001
Hepatitis C Virus	94	48/46	50.3 ± 15.4(18-86)	474.9 ± 123.1	<0.001
Other viruses*	176	124/52	12.9 ± 19.1(1-75)	251.2 ± 256.3	>0.05
Microspironema pallidum	42	24/18	37.2 ± 16.1(1-69)	365.8 ± 476.6	<0.001
Canidia albicans	14	11/3	66.2 ± 22.0(0.2-90)	285.0 ± 215.3	>0.05
Healthy controls	141	81/60	44.0 ± 11.4(22-70)	168.5 ± 56.7	

*: including herpes simplex virus, Coxsackie virus, Epstein-Barr virus, cytomegalovirus, hepatitis A virus and hepatitis E virus.

**Figure 5:**
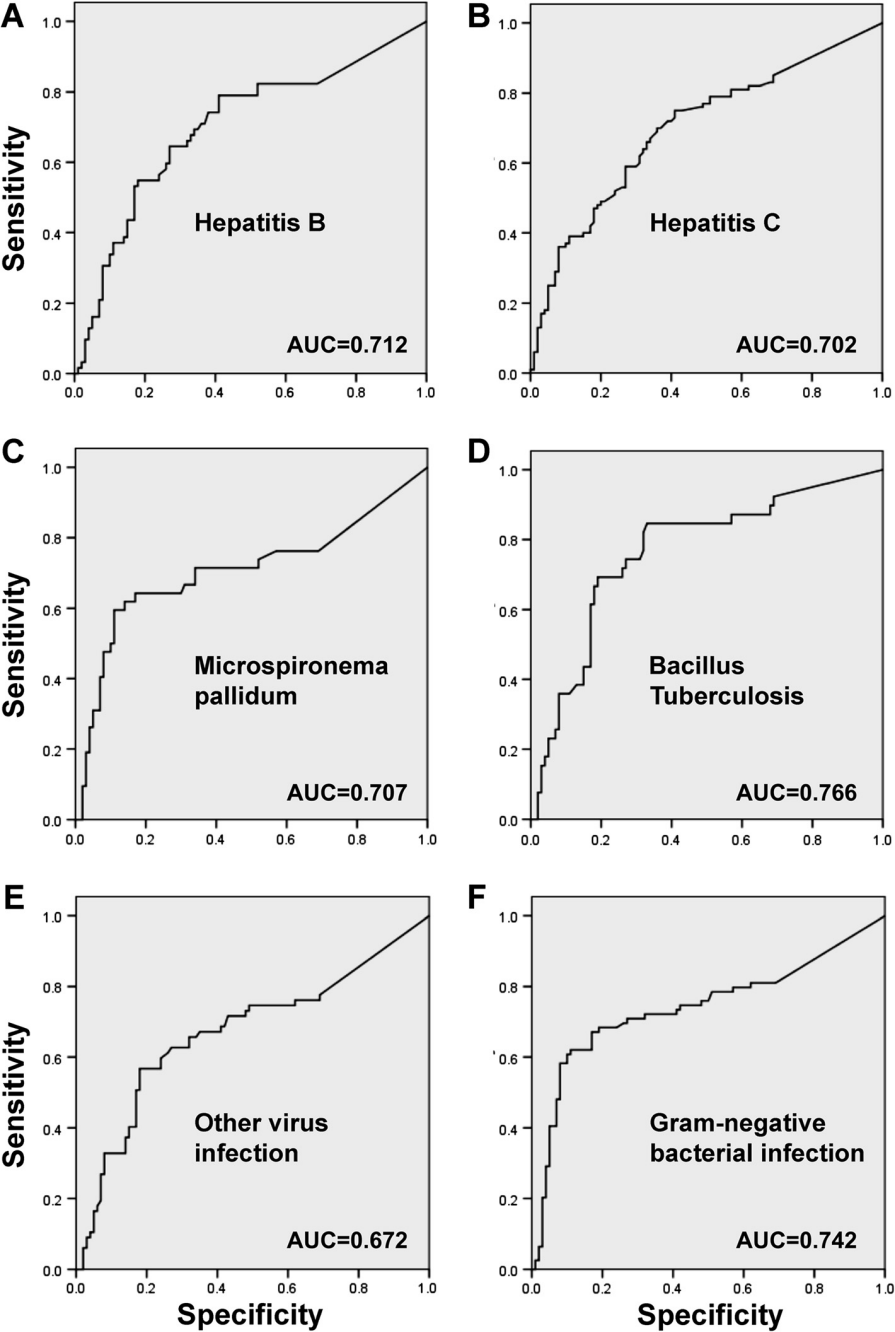
**Receiver operating characteristic (ROC) curves illustrating the diagnostic impact of serum sMICA levels in different infectious diseases, with the values of area under the ROC curve (AUC) labeled for each type of infections.** The data indicate that test has an intermediate diagnostic impact (AUC > 0.7) among patients with infections by hepatitis B (**A**) and C (**B**) viruses; Microspironema pallidum (**C**), bacillus tuberculosis (**D**) and Gram-negative bacterium (**F**). In contrast, the diagnostic value is low in the patient group with infections including herpes simplex virus, Coxsackie virus, Epstein-Barr virus, cytomegalovirus, hepatitis A virus and hepatitis E virus (**E**).

As with the cancer disease groups, the diagnostic values of sMICA in the infection disease groups were assessed by ROC curve fitting. The AUC value for hepatitis B was 0.712, for hepatitis C was 0.701, syphilis helicoid, 0.707, tubercle bacilli, 0.766, gram-negative bacilli, 0.742, and for other virus infections, 0.672 (Figure 5A-F).

## Discussion

Previous association studies have shown a link of polymorphism of MICA/B and/or alteration in sMICA/B to cancers, autoimmunity and host-graft reaction [11,17,19,25,26,34-36]. Serum sMICA/B levels are potential biomarkers of pathogenic implications for these disease conditions. For instance, upregulation of sMICA/B may be involved in the escape of tumor cells from immune cell attack and clearance [1-3,19,26,27]. Accordingly, inhibition of sMICA/B production and/or preventing their shedding from cell surface may be of therapeutic potential in treating these human diseases [24,37]. A better understanding of sMICA/B alteration in a broad disease spectrum is of important clinical relevance. Using a relatively large sample pool, we show here elevations of serum sMICA in association with infectious diseases in addition to cancers in a southern Chinese Han population.

Elevations of serum sMICA and sMICB have been shown in a number of malignant diseases, with sMICA potentially being more suitable than sMICB for early diagnosis of some cancers [33,34]. Specifically, lung, breast, digestive system (hepatic, pancreas, gastric, colorectal) and urological cancers (prostate and renal) are associated with significant increase of serum sMICA relative to healthy controls as well as benign tumors. Consistent with these reports, the present study finds elevation of serum sMICA among Chinese patients with several types of malignant tumors, which are, or are approaching to, statistical significance relative to controls. In particular, the elevation appears to be especially dramatic in the hepatic cancer patients. The present data also suggest a certain extent of correlation of sMICA levels to the clinical stages of hepatic cancer. Thus, a significant difference exists between T2, T3 and T4 as compared to T1 stage cases, as well as between T2 and T3 stage patients. Somewhat surprising, the levels of sMICA are reduced in T4 compared with T3 stage patients in our studied cohort. One possible explanation for this finding may be that the lowering of sMICA in the T4 stage group occurs as a result of severe systematic or hepatic deficiency in cell function, such as failure of protein synthesis, among end-stage cancer patients. The ROC analysis in the present study reveals moderate diagnostic value for liver cancer, but marginal impacts for other cancer types. Taken together, serum sMICA and ROC analyses in the present study suggest a trend of elevation of the protein in liver, lung and laryngeal carcinomas in Chinese population, with the laboratory test being of significant value in the diagnosis and prognosis of hepatic cancer.

Evidence suggests that infections by microbiological pathogens may alter the expression and functionality of membrane bound MICA/B [2,4,16,36]. For instances, changes in MICA/B and/or NKG2D levels and their activities are associated with infections caused by human cytomegalovirus [28], hepatitis B and C viruses [14,29], herpes simplex virus [30], human immunodeficiency virus [31] and vesicular stomatitis virus [46]. Sex-transmitted *Chlamydia trachomatis* may also down-regulate MICA/B expression on cell membrane [16,44,50]. However, whether infections caused by microbiological pathogens also affect serum sMICA levels remains poorly understood.

The present study provides evidence that serum sMICA levels are elevated in various infectious diseases by microbiological pathogens. In detail, sMICA levels are increased to above 2 fold in bacterial infections with enterobacteriaceae, mycobacterium tuberculosis, Gram-positive cocci, non-fermenting Gram-negative bacteria and Microspironema pallidum. Among virus infectious diseases, sMICA levels are significantly increased in hepatitis B and C. However, we fail to find significant elevations of sMICA among patients infected by herpes simplex virus, Coxsackie virus, Epstein-Barr virus, cytomegalovirus, hepatitis A virus and hepatitis E virus, and by the Canidia albicans fungus. These data implicate that increased shedding of membrane MICA molecules presumably from infected/inflammatory cells may occur in several types of infectious diseases, which may lead to the observed rise of sMICA in serum. Given the findings of the elevation in many but not all infectious diseases, one may hypothesize that the extent of sMICA elevation could be potentially relevant to either the pathogens, or alternatively, the amount of involvement of infected cells in the body. Our ROC analyses suggest that serum sMICA measurement appears to have an intermediate diagnostic value for infections with hepatitis B and C virus, Microspironema pallidum, tuberculosis and Gram-negative bacteria (i.e., AUC > 0.7).

## Conclusions

The present study shows elevation of serum sMICA levels in patients suffering from several types of malignant and infectious diseases relative to healthy controls in a southern China population. The data suggest that serum sMICA is of potential diagnostic value for some bacterial and viral infections, in addition to malignant disorders as reported previously. Based on our currently findings, microbiological infections should be considered as a part of differential diagnosis while evaluating serum sMICA changes in other disease conditions.

## Competing interests

The authors declare that they have no competing interests.

## Authors’ contributions

XJ, PY and RC designed the experiment. XJ, ZH, QZ, YJ, XW, YL and GJ collected the samples and performed experiments. XJ, JH, LZ and XXY analyzed data. XJ and XXY wrote the paper. All authors read and approved the final manuscript.
